# Supplementary material for: Execution and Design of an Anti HPIV-1 Vaccine with Multiple Epitopes Triggering Innate and Adaptive Immune Responses: An Immunoinformatic Approach
Source: Vaccines (Basel). 2022 May 29;10(6):869. doi: 10.3390/vaccines10060869 (PMC9228812; doi:10.3390/vaccines10060869)
Supplement: Supplementary file 1 [file vaccines-10-00869-s001.zip › vaccines-1672594-supplementary.pdf]

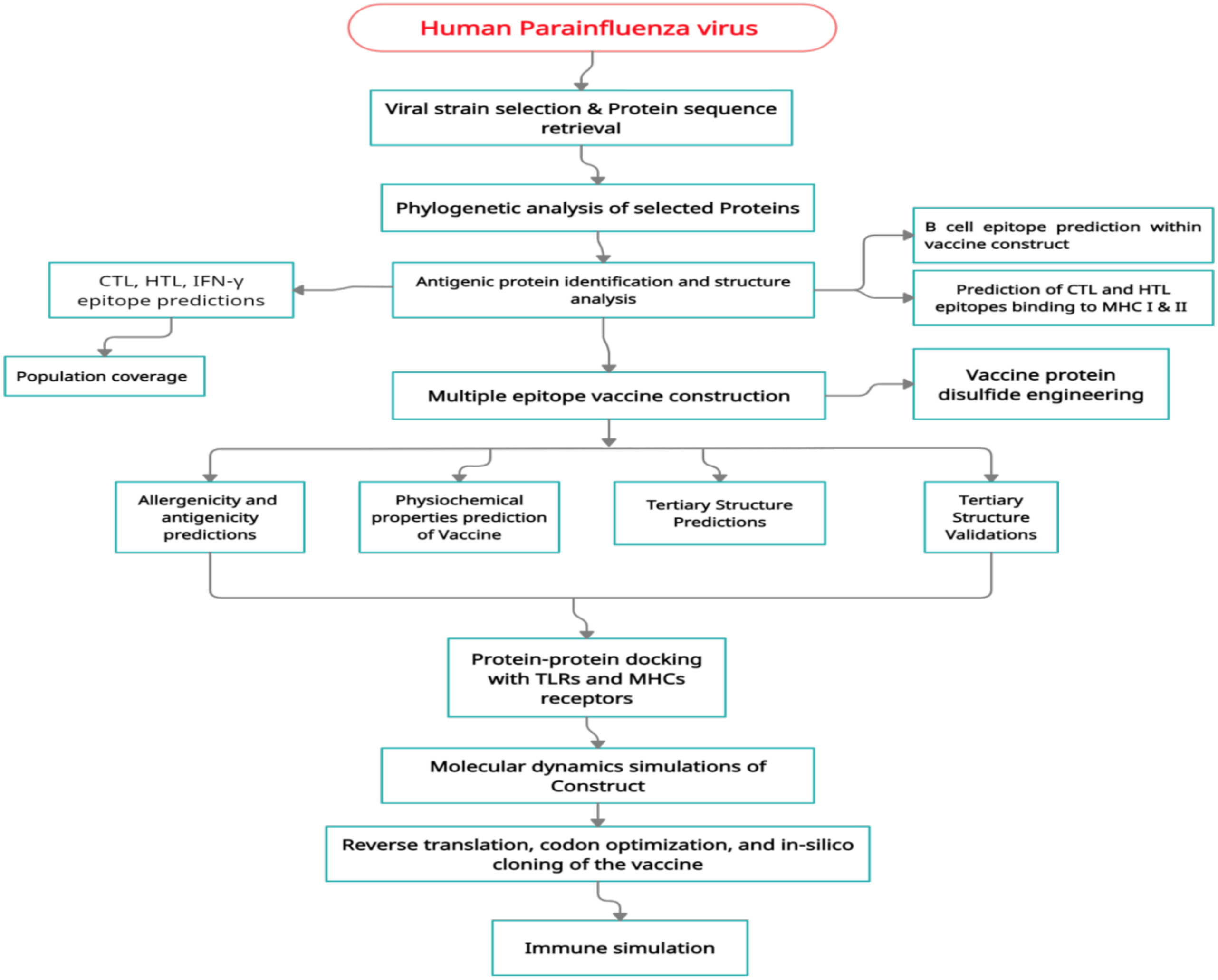

Figure S1: Flow Chart of Methodology.

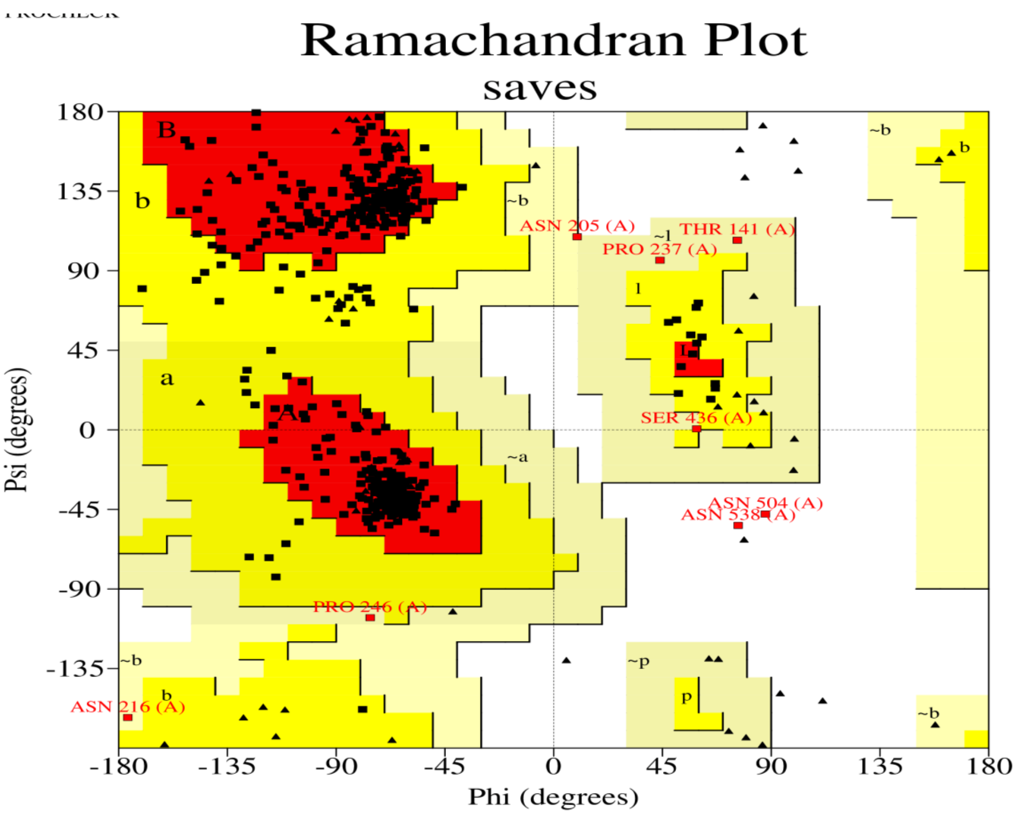

Plot statistics

Residues in most favoured regions [A,B,L]	478	90.5%
Residues in additional allowed regions [a,b,l,p]	44	8.3%
Residues in generously allowed regions [~a,~b,~l,~p]	4	0.8%
Residues in disallowed regions	2	0.4%
	----	------
Number of non-glycine and non-proline residues	528	100.0%
Number of end-residues (excl. Gly and Pro)	2	
Number of glycine residues (shown as triangles)	62	
Number of proline residues	49	

Total number of residues	641	

Based on an analysis of 118 structures of resolution of at least 2.0 Angstroms and R-factor no greater than 20%, a good quality model would be expected to have over 90% in the most favoured regions.

Figure S2: Ramachandran plot predicted by the PROCHECK using the refined model of vaccine, the score is above 90 showing that the 3-D model is valid

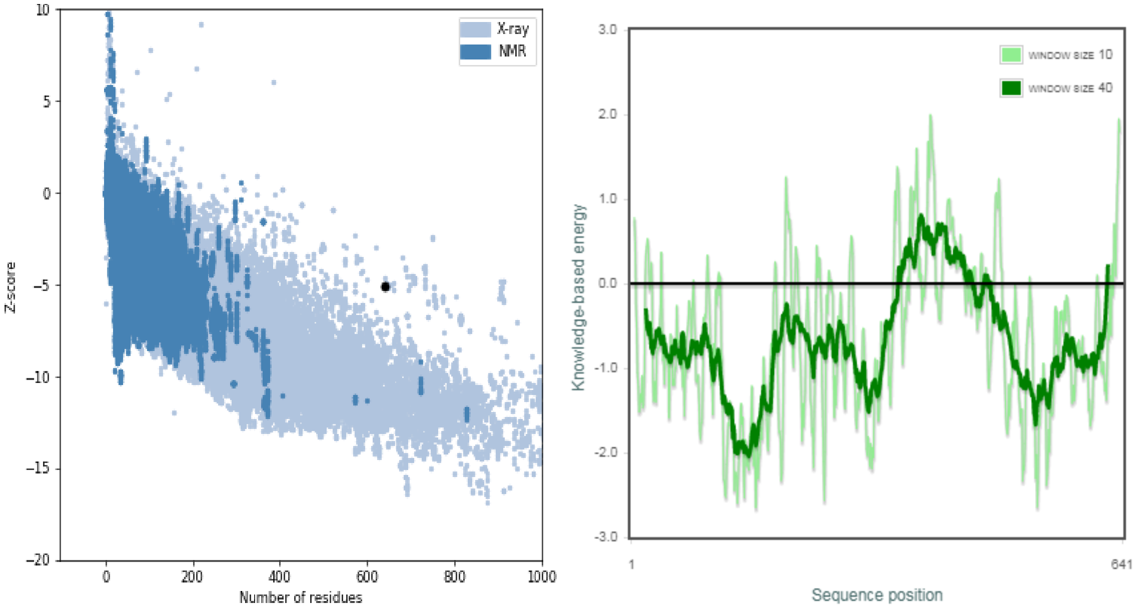

Figure S3: Graphical representation of Z-score calculation of Peptide compared with the native protein energies, the negative 5.06 z-score is another proof of the validation of 3D model of the vaccine construct

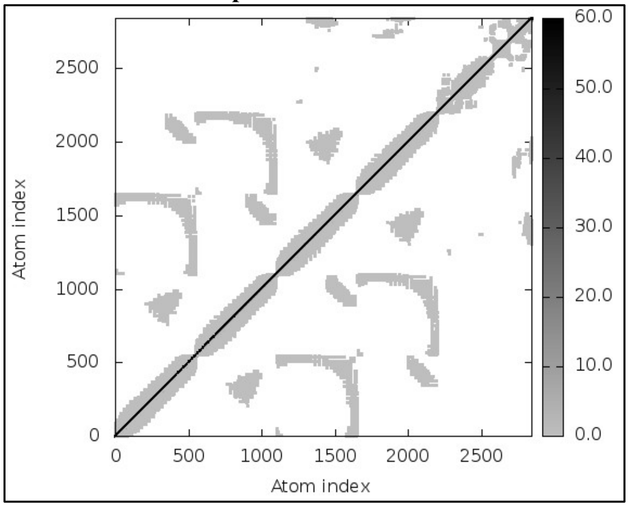

Figure S4: Above diagrams shows 3-D model, B-factor/Mobility, Eigenvalues, Variance, Covariance map, and Elastic network of TLR-2 Complex respectively from left to right

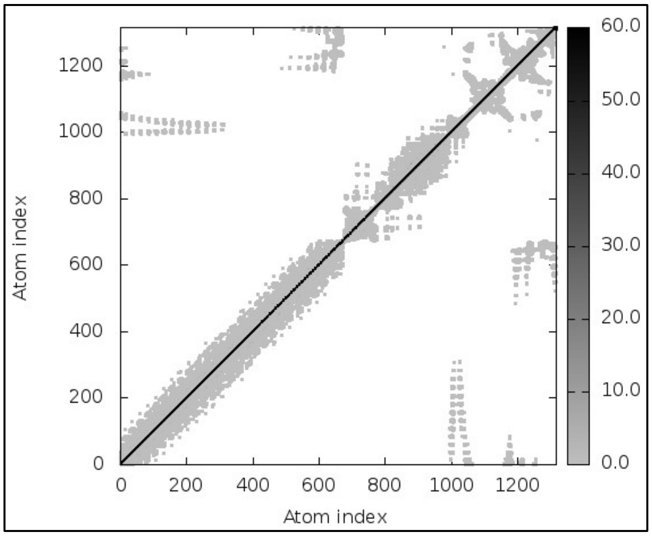

Figure S5: Above diagrams shows 3-D model, B-factor/Mobility, Eigenvalues, Variance, Covariance map, and Elastic network of TLR-3 Complex respectively from left to right

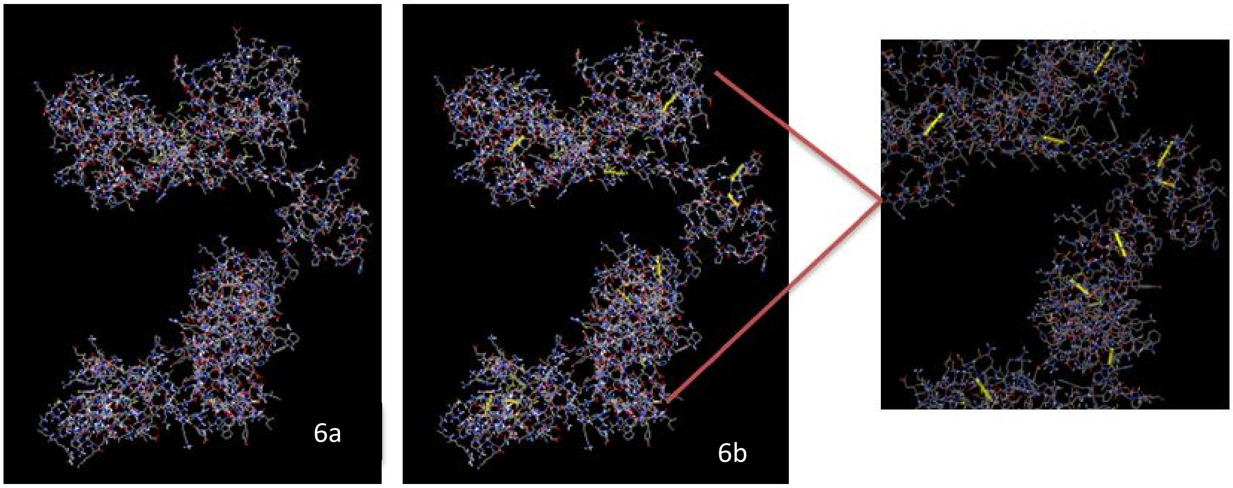

Figure S6: Wireframe structure of real and mutated model. 6a) original structure of model 6b) Mutated model showing the disulfide bonds (yellow) between the selected pairs of residues

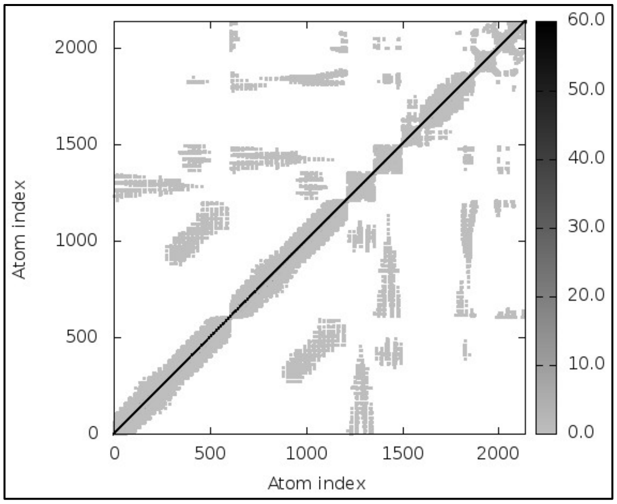

Figure S7: Above diagrams shows 3-D model, B-factor/Mobility, Eigenvalues, Variance, Covariance map, and Elastic network of TLR-4 Complex respectively from left to right

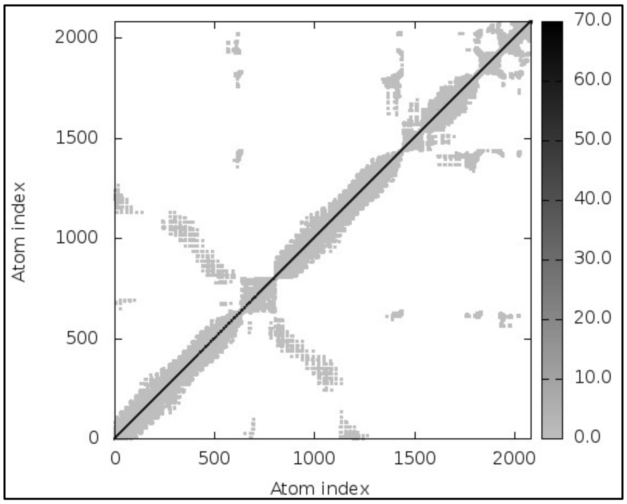

Figure S8: Above diagrams shows 3-D model, B-factor/Mobility, Eigenvalues, Variance, Covariance map, and Elastic network of TLR-5 Complex respectively from left to right

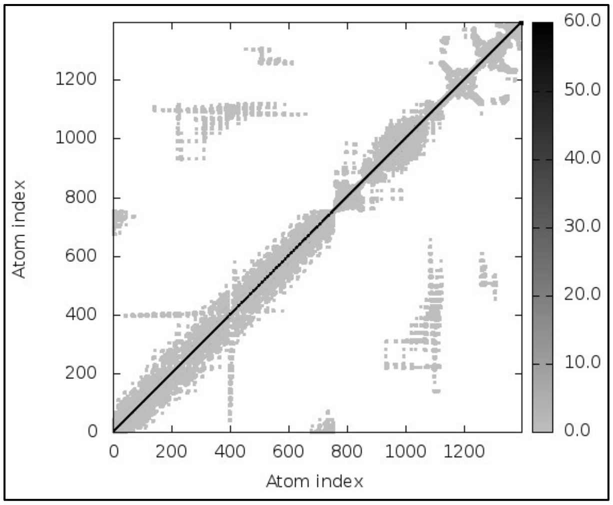

Figure S9: Above diagrams shows 3-D model, B-factor/Mobility, Eigenvalues, Variance, Covariance map, and Elastic network of TLR-8 Complex respectively from left to right

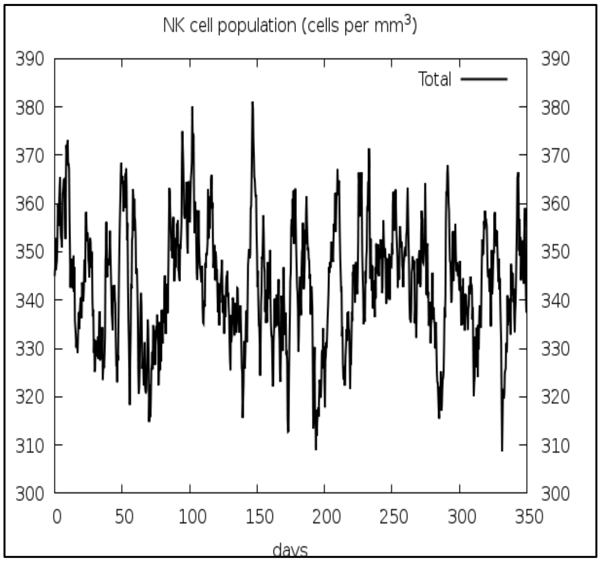

Figure S10: Graph showing Natural Killer (NK) cells production pattern

Table S1: VaxiJen score of five selected proteins along with their UniProt IDs.

UniProt ID	Protein	Vaxijen 2.0 score
**P12605**	Fusion glycoprotein (F)	0.5106
**Q01427**	Membrane (M) protein	0.4895
**P16071**	**Hemagglutinin-neuraminidase (HN) protein**	**0.5343**
**J7FXM0**	Nucleocapsid (N) protein	0.4816
**P26590**	Nucleoprotein	0.5060

Table S2: All The B-Cell epitopes predicted by the IEDB server along with the length of each epitope.

No.	Start	End	Peptide	Length
**1**	**4**	**32**	**KGKTNSSYWSTTRNDNSTVNTYIDTPAGK**	**29**
**2**	**63**	**71**	**DTCMKTNIM**	**9**
**3**	**78**	**97**	**ESAKTIKETITELIRQEVIS**	**20**
4	116	116	K	1
5	118	190	SRDLTQLIEKSCNRQELAQICENTIAIHHADGISPLDPHDFWRCPV GEPLLSNNPNISLLPGPSLLSGSTTIS	73
6	216	221	CADIGK	6
7	234	238	NSDMY	5
8	249	254	YDINDN	6
**9**	**275**	**287**	**TVNETTDYSSEGI**	**13**
**10**	**301**	**313**	**TKSHRYKNEDITF**	**13**
**11**	**343**	**375**	**TTPLQGDTKCVTNRCANVNQSVCNDALKITWLK**	**33**
12	389	393	YLSDR	5
13	403	404	IT	2
14	429	433	GWHSH	5
**15**	**445**	**470**	**MTIKWAPHEVLSRPGNQDCNWYNRCP**	**26**

No.	Start	End	Peptide	Length
16	499	503	NTSRV	5
17	516	516	I	1
**18**	**520**	**528**	**RLKNVQLEA**	**9**
19	554	557	SLNT	4

Table S3: Reference set alleles for MHC-1 and MHC-II molecules.

MHC-1 ALLELES			
**Alleles**	**length**	**Alleles**	**length**
HLA-A*01:01	9	HLA-A*33:01	10
HLA-A*01:01	10	HLA-A*68:01	9
HLA-A*02:01	9	HLA-A*68:01	10
HLA-A*02:01	10	HLA-A*68:02	9
HLA-A*02:03	9	HLA-A*68:02	10
HLA-A*02:03	10	HLA-B*07:02	9
HLA-A*02:06	9	HLA-B*07:02	10
HLA-A*02:06	10	HLA-B*08:01	9
HLA-A*03:01	9	HLA-B*08:01	10
HLA-A*03:01	10	HLA-B*15:01	9
HLA-A*11:01	9	HLA-B*15:01	10
HLA-A*11:01	10	HLA-B*35:01	9
HLA-A*23:01	9	HLA-B*35:01	10
HLA-A*23:01	10	HLA-B*40:01	9
HLA-A*24:02	9	HLA-B*40:01	10
HLA-A*24:02	10	HLA-B*44:02	9
HLA-A*26:01	9	HLA-B*44:02	10
HLA-A*26:01	10	HLA-B*44:03	9
HLA-A*30:01	9	HLA-B*44:03	10
HLA-A*30:01	10	HLA-B*51:01	9
HLA-A*30:02	9	HLA-B*51:01	10
HLA-A*30:02	10	HLA-B*53:01	9
HLA-A*31:01	9	HLA-B*53:01	10
HLA-A*31:01	10	HLA-B*57:01	9
HLA-A*32:01	9	HLA-B*57:01	10
HLA-A*32:01	10	HLA-B*58:01	9
HLA-A*33:01	9	HLA-B*58:01	10
**MHC-II ALLELES**			
**Alleles**		**length**	

**HLA-DRB1*03:01**	15
**HLA-DRB1*07:01**	15
**HLA-DRB1*15:01**	15
**HLA-DRB3*01:01**	15
**HLA-DRB3*02:02**	15
**HLA-DRB4*01:01**	15
**HLA-DRB5*01:01**	15

Table S4: Scores of different parameters used by GalaxyRefine for the refinement of 3-D model.

GDT-HA	RMSD	MolProbity	Clash score	Poor rotamers	Rama favored
0.9844	0.286	2.287	25.0	0.6	94.1

Table S5: List of 12 selected residue pairs for disulfide engineering, all the selected pairs have bond energy less than 2.20 kcal/mol.

	Residue 1			Residue 2			Bond	
	**Chain**	**Seq #**	**AA**	**Chain**	**Seq #**	**AA**	χ_3	**kcal/mol**
**1**	A	183	GLY	A	210	GLY	-88.43	0.38
**2**	A	510	ALA	A	543	ALA	-100.70	1.20
**3**	A	192	ILE	A	204	ASP	+95.57	1.40
**4**	A	448	ASN	A	474	ALA	-95.94	1.46
**5**	A	581	ASP	A	584	TYR	+99.43	1.53
**6**	A	17	CYS	A	27	LEU	+89.31	1.71
**7**	A	261	ILE	A	275	LYS	+91.37	1.74
**8**	A	325	PRO	A	358	VAL	+98.17	1.76
**9**	A	28	SER	A	46	CYS	+107.08	1.77
**10**	A	295	PRO	A	299	ALA	-80.80	1.92
**11**	A	45	LYS	A	79	CYS	+116.31	1.99
**12**	A	326	GLY	A	330	ALA	+72.74	2.17

Table S6: Maximum ClusPro energies, cluster members and patchdock of all the TLRs used for Molecular docking.

TLRs	TLR 2	TLR 3	TLR 4	TLR 5	TLR 8
**ClusPro2.0 (kcal/mol)**	-1144.3	-1202.9	-1169.7	-1441.3	-1320.7
**Cluster Members**	51	29	43	40	54
**PatchDock Score**	18882	18696	20564	23004	15182
